# Analytical Geometry Description, Clinical Node Distribution, and Geometric Performance of ZAP-X and Its Comparison With Gamma Knife

**DOI:** 10.7759/cureus.89112

**Published:** 2025-07-31

**Authors:** Borja Aguilar, Vânia Dias, Enrique Pascual, Leonora Trinidad, Carlos Rodrigues

**Affiliations:** 1 Medical Physics Department, Mercurius Health, Lisbon, PRT; 2 Radiation Oncology Department, Institute of Advanced Radiosurgery (IRCA), Hospital Viamed Santa Elena, Madrid, ESP; 3 Radiation Oncology Department, Mercurius Health, Lisbon, PRT

**Keywords:** geometric efficiency, geometric performance, grs, radiosurgery, srs, zap-x

## Abstract

In radiotherapy, it is increasingly common to find specialized devices in different treatment techniques. The ZAP-X is a radiosurgery system which features a sophisticated and complex geometric design that allows to deliver a high dose of precisely targeted radiation while minimizing the radiation effect on the surrounding normal brain tissue.

Its combination of yoked gimbals allows a maximum theoretical geometric efficiency of $2\sqrt{2} \pi$ steradian solid angle coverage, which means that it is possible to cover 71% of the surface around the patient's head, making the ZAP-X the commercially available radiosurgery system with the largest beam coverage.

While irradiation is possible from any angle in the Treatment Delivery System (TDS), collision-free space is limited in the Treatment Planning System (TPS), so the geometric performance that the ZAP-X can achieve in clinical practice is different and smaller than the theoretical geometric efficiency of the system. However, it has greater geometric efficiency (71%) and higher clinical geometric performance (39%) than the Gamma Knife system (31%).

A general analytical geometric description of the gyroscopic system is provided, analyzing the movement and the different combinations of positions of its gantries to finally calculate its geometric efficiency, both from a theoretical and clinical point of view, and compare it with the Gamma Knife system.

## Introduction

In radiotherapy, it is increasingly common to find specialized devices in different treatment techniques. In this sense, different specific solutions for intracranial stereotactic radiosurgery (SRS) have been developed, the most innovative system being the ZAP-X.

The ZAP-X is a radiosurgery system, also called a gyroscopic radiosurgery system due to the way its components move, which features a sophisticated and complex geometric design that allows all the components and systems necessary to safely perform external radiotherapy treatments to be integrated into a very small space [1-3]. It allows for the delivery of a high dose of precisely targeted radiation using highly focused three FFF X-ray beams to a specific area of the brain to treat a tumor or another abnormality while minimizing the radiation effect on the surrounding normal brain tissue. To achieve this goal, a novel gyroscopic radiosurgery platform has been developed.

It is common to compare different systems, their response, clinical results, and performance [4,5]. The purpose of this work is to calculate and evaluate the geometric efficiency and the clinical geometric performance of a ZAP-X gyroscopic frameless radiosurgery system (GRS) by analyzing the treatment delivery reports of all treatments delivered over the course of more than two years and compare the result with Gamma Knife, the other system available on the market dedicated exclusively to intracranial radiosurgery.

## Technical report

General geometric description of the gyroscopic system

**Figure 1:**
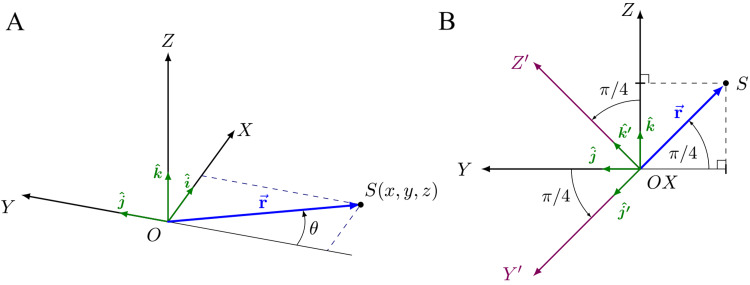
Coordinate system used to define the oblique angle (A) Definition of the oblique angle: θ. (B) Oblique plane, X'Y', and oblique axis, Z'

Let us suppose that the point $S$ is a representative point of the system, for example, the linear accelerator (LINAC), the collimator wheel or the radiation source, and that its movement is restricted to a circle of radius $r$ in the $XY$ plane, which has its center at point $O$, the isocenter and the origin of the Cartesian coordinate system $XYZ$ (Figure 1, left). The Cartesian coordinate system $XYZ$ is solidary to the treatment room, and the direction and sense of the axes coincide with those of the ZAP-X table. The axial axis coincides with the $Y$​​​ axis and is aligned along the long axis of the system. We will use polar coordinates to describe the position of the point $S$ in the $XY$ plane. The vector $\vec{r}$ is the vector that joins the point $O$, the isocenter, with the MV X-ray source. The modulus of $\vec{r}$, $r=\lvert \vec{r} \lvert$, is 450 mm, and theta, $\theta$, represents the angle that we will call oblique. The projection of point $S$ on the $X$, $Y$, and $Z$ axes is given by

\begin{align} \vec{x}&= r\sin\theta \, \hat{i} \\ \vec{y}&= -r\cos\theta \, \hat{j} \\ \vec{z}&= 0 \,\hat{k} \end{align}

where $\hat{i}$, $\hat{j}$, and $\hat{k}$ are the unitary vectors on the $X$, $Y$, and $Z$ axes, respectively.

We now create a new reference frame by rotating the $XYZ$ reference frame 45° in the negative direction (according to the right-hand grip rule) of the $X$ axis, since this is the angle formed by the axis of the two gantries of the ZAP-X system [6]. As a result, we obtain the reference frame $X'Y'Z'$​​​​ (Figure 1, right). Point $S$, which moves according to the rotation, will be contained in the $X'Y'$ plane for all $\theta$ angle values. The plane $X'Y'$ represents the oblique plane, which is perpendicular to the oblique axis defined by the $Z'$ axis. Thus, the coordinates of point $S$ in the $X'Y'Z'$​​​​​​​ coordinate systems will now be expressed as

\begin{align} \vec{x'}&= r\sin\theta\, \hat{i'}\\ \vec{y'}&= -r\cos\theta\, \hat{j'}\\ \vec{z'}&= 0 \,\hat{k'} \end{align}

where $\hat{i'}$​​​​​​​, $\hat{j'}$​​​​​​​, and $\hat{k'}$​​​​​​​ are the unitary vectors on the $X'$​​​​​​​​​​​​​​, $Y'$,​​​​​​​​​​​​​​ and $Z'$​​​​​​​​​​​​​​ axes, respectively.

Since the rotation is around the $X$​​​​​​​ axis, the projection of point $S$​​​​​​​ on the $Y'Z'$​​​​​​​ plane will always move on the $Y'$​​​​​​​ axis, and its modulus will be given by $\lvert\vec{y'}\rvert = r\cos\theta$​​​​​​​. The coordinates of point $S$​​​​​​​ in the $XYZ$​​​​​​​ coordinate system will now be expressed as

\begin{align} \vec{x}&= \vec{x'}\\ \vec{y}&= - \lvert\vec{y'}\rvert\cos(\pi/4)\, \hat{j} \\ \vec{z}&= \lvert\vec{y'}\rvert\sin(\pi/4)\, \hat{k} \end{align}

and substituting $\lvert\vec{y'}\rvert = r\cos\theta$​​​​​​​​​​​​​​,

\begin{align} \vec{x}&= \vec{x'} = r\sin\theta\, \hat{i}\\ \vec{y}&= -\lvert\vec{y'}\rvert\cos(\pi/4)\, \hat{j} = - r\cos\theta\cos(\pi/4)\, \hat{j}\\ \vec{z}&= \lvert\vec{y'}\rvert\sin(\pi/4)\, \hat{k} = r\cos\theta\sin(\pi/4)\, \hat{k} \end{align}

**Figure 2:**
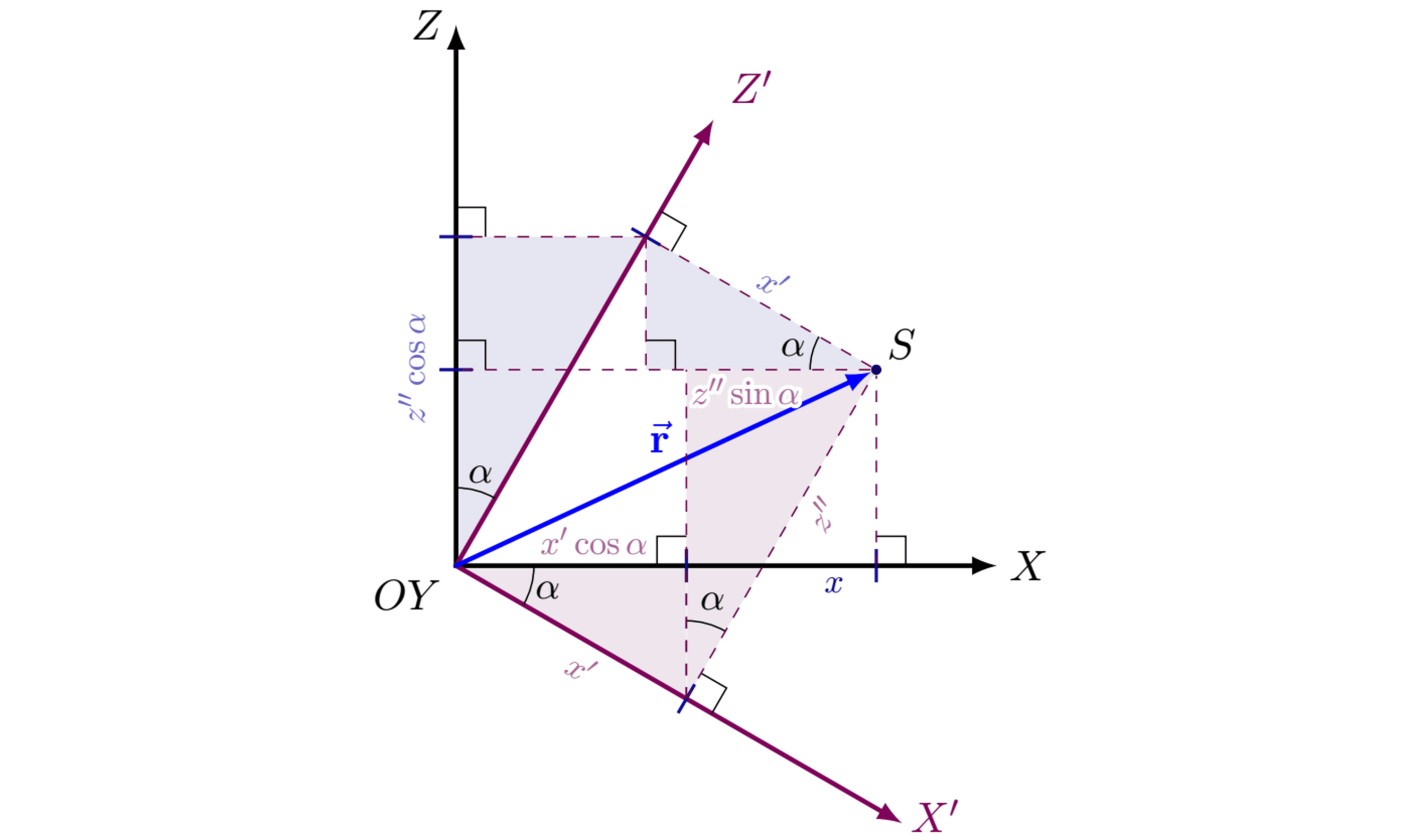
Axial angle, α, axial plane, XZ, and axial axis, Y. Y' axis is not represented for the sake of simplicity

**Figure 3:**
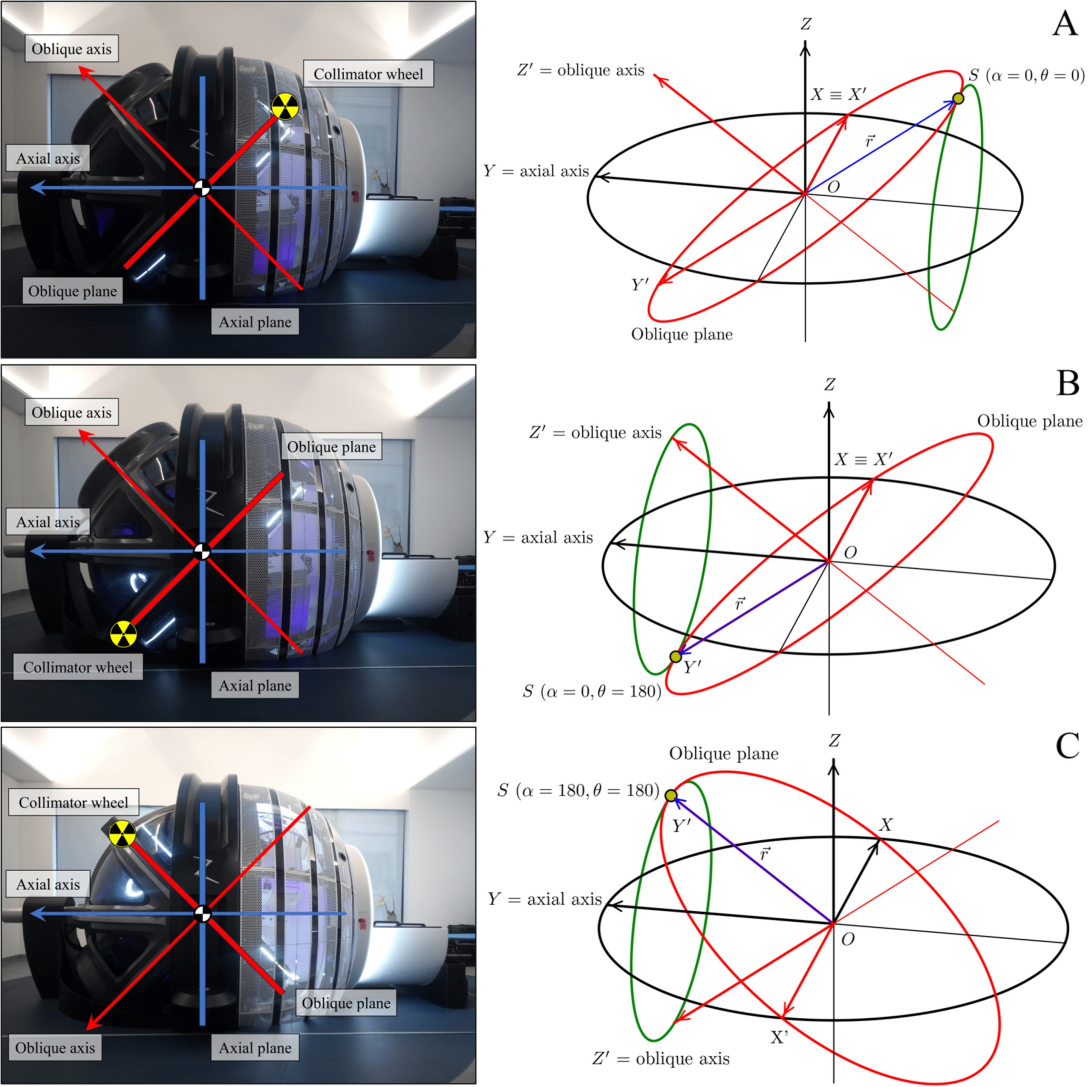
Different positions of the axial and oblique gantries (A) The axial gantry is at 0° and the oblique gantry is at 0°. The radiation source is in the most anterior and caudal position. The XZ plane represents the axial plane. The path of the source with the axial angle is shown in green. (B) From the previous position (A), the radiation source rotates 180° around the oblique axis, Z'. Now the axial gantry is at 0° and the oblique gantry is at 180°. (C) From the previous position (B), the source, attached to the oblique plane, rotates around the axial axis 180°. Now the axial gantry is 180°, and the oblique gantry is 180°. This is the Home position

The last transformation is a rotation by an angle $\alpha$​​​​​​​​​​​​​​ around the $Y$​​​​​​​ axis (Figure 2). We will call this angle axial. This rotation is in the positive direction (according to the right-hand grip rule) of the $Y$​​​​​​​​​​​​​​ axis, also called the axial axis, and will modify the $x$​​​​​​​ and $z$​​​​​​​ coordinates, leaving the $y$​​​​​​​ coordinate invariant. The $XZ$​​​​​​​​​​​​​​ plane represents the axial plane (Figure 3).

The rotation matrix around the $Y$​​​​​​​ axis by an angle $\alpha$​​​​​​​ will be $R_Y(\alpha)$​​​​​​​​​​​​​​

\begin{align} R_Y(\alpha)= \begin{pmatrix} \cos\alpha & 0 & -\sin\alpha \\ 0 & 1 & 0 \\ \sin\alpha & 0 & \cos\alpha \end{pmatrix} \end{align}

If this transformation is applied to the previous system of equations, we can get the position of point $S$​​​​​​​ in the $XYZ$​​​​​​​ coordinate system

\begin{align} \vec{x}&= (x'\cos\alpha + z''\sin\alpha)\hat{i} = \left(r\sin\theta\cos\alpha + r\cos\theta\sin(\pi/4)\sin\alpha \right)\hat{i}\\ \vec{y}&= \vec{y'} = -r\cos\theta\cos(\pi/4)\hat{j}\\ \vec{z}&= (-x'\sin\alpha + z''\cos\alpha)\hat{k} = \left(-r\sin\theta\sin\alpha + r\cos\theta\sin(\pi/4)\cos\alpha\right)\hat{k} \end{align}

where $z''$​​​​​​​ is the projection of point $S$​​​​​​​ in the $Z$​​​​​​​ axis before the transformation. Simplifying

\begin{align} \vec{x} &= r \left(\sin\theta\cos\alpha + \frac{\sqrt{2}}{2}\cos\theta\sin\alpha \right) \, \hat{i}\\ \vec{y} &= -r \frac{\sqrt{2}}{2}\cos\theta \, \hat{j}\\ \vec{z} &= r \left( \frac{\sqrt{2}}{2}\cos\theta\cos\alpha - \sin\theta\sin\alpha \right) \, \hat{k} \end{align}

These equations allow the user to obtain the position of the source, $(x,y,z)$​​​​​​​, in the $XYZ$​​​​​​​ reference frame as a function of the oblique and axial angles, $(\theta,\alpha)$​​​​​​​, respectively. It can be verified that

\begin{align} \lvert \vec{x}\rvert &\in \left[-r, r\right]\\ \lvert \vec{y}\rvert &\in \left[-\sqrt{2}\,r/2,\sqrt{2}\,r/2\right]\\ \lvert \vec{z}\rvert &\in \left[-r,r\right] \end{align}

Finally, and as a summary of all the above, the position $(x,y,z)$​​​​​​​ of point $S$​​​​​​​ can be obtained as

\begin{align} \vec{r} = (x,y,z) = (r\sin\theta,-r\cos\theta,0) \ R_X(-\pi/4) \ R_Y(\alpha) \end{align}

where $R_X(-\pi/4)$​​​​​​​ is the rotation matrix around the $X$ axis, an angle $-\pi/4$​​​​​​​, substituting

\begin{align} \vec{r}= (r\sin\theta, -r\cos\theta,0) \begin{pmatrix} 1 & 0 & 0 \\ 0 & \cos(\pi/4) & -\sin(\pi/4)\\ 0 & \sin(\pi/4) & \cos(\pi/4) \end{pmatrix} \begin{pmatrix} \cos\alpha & 0 & -\sin\alpha\\ 0 & 1 & 0 \\ \sin\alpha & 0 & \cos\alpha \end{pmatrix} \end{align}

Singular positions of the gantry, isocenter, and head center

Now, we can try different combinations of the axial and oblique angles (Figure 3). The singular positions, which we will call cardinal directions, are listed in Table 1. The "Home" position is the system's resting position and where the gantries must be located to automatically insert and extract the treatment couch. All treatments start and end at this gantry's position.

**Table 1:** Different combinations of the axial and oblique angles and the corresponding coordinates of the radiation source in the XYZ frame

Description	Axial angle, α	Oblique angle, θ	X	Y	Z
Home	180	180	0	√2r/2	√2r/2
North pole	270	90	0	0	r
Patient left	0	90	r	0	0
South pole	90	90	0	0	-r
Patient right	180	90	-r	0	0

**Figure 4:**
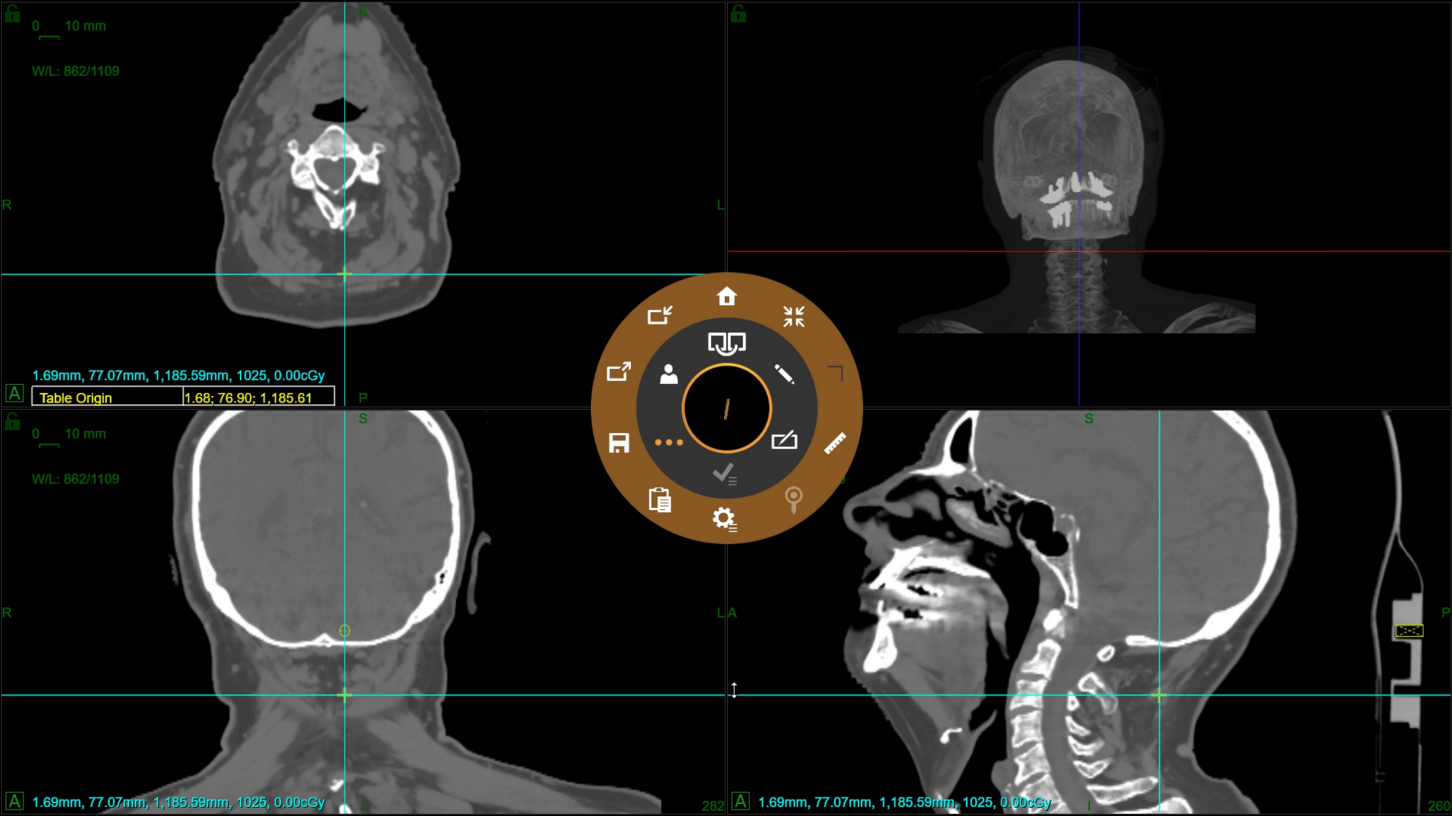
Head center position (table origin) marked with a green cross on the TPS. The blue crosshair intersects approximately at the head center. The couch top geometry is used to automatically position the head center. TPS: Treatment Planning System

The isocenter is the point around which the axial and oblique gantries rotate. The system has a characteristic point called "head center" (or table origin), which is defined as the virtual point on the treatment couch that lies at the isocenter when the treatment couch is in the $(0,0,0)$ position. The system is calibrated to meet this condition. The "head center" is the point used to define the extent of the dose calculation matrix and is automatically determined by the TPS in the simulation CT scanner image set from the couch top geometry (Figure 4). Therefore, no stereotaxic frames, lasers, or external markers are required during the simulation process, simplifying the radiotherapy workflow.

Nodes, paths, path density, and trajectories

**Figure 5:**
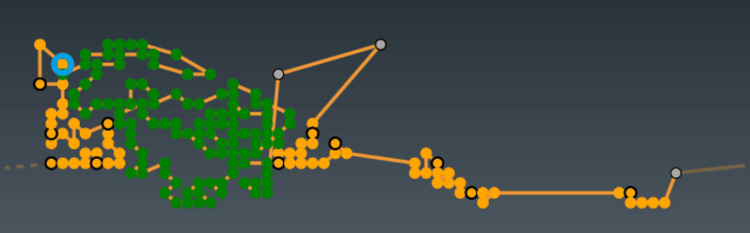
Trajectory followed by the radiation source and path progress displayed on the TDS during the treatment delivery. The horizontal and vertical axis are the axial and oblique gantry locations, respectively (Figure 6)

**Figure 6:**
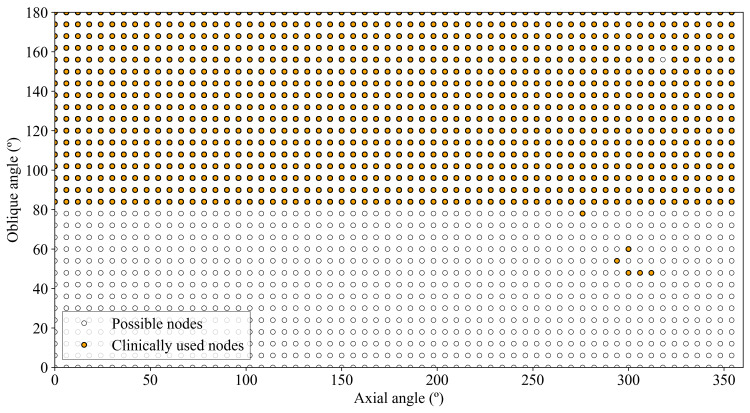
The figure shows the possible nodes and those that have been used clinically. In total, 1026 have been used. The oblique angle of 156º has not used the axial gantry of 318º. Nodes with an oblique gantry angle less than 84° have only been used once

The ZAP-X uses a step-and-shoot beam delivery technique. Therefore, the beam is switched off while the gantries are moving. Each of the stopping points from which radiation is delivered is called a node (Figures 5, 6). The trajectory is the path followed by the radiation source to deliver all the nodes associated with the same collimator. Trajectories are calculated by the TPS using an algorithm to determine the appropriate path to minimize the delivery time of each collimator. During the treatment delivery, the trajectories can be visualized on the Treatment Delivery System (TDS) (Figure 5).

Trajectories with a constant oblique angle, $\theta=$​​​​​​​constant, are known as paths and describe circles in the $XZ$​​​​​​​ plane (Figure 3) with coordinate $\vec{y}=-r\sqrt{2}/2\cos\theta\, \hat{j}$. The radius of this circular path will be given by

\begin{align} \sqrt{{\lvert\vec{x} \rvert}^2 + {\lvert\vec{z}\rvert}^2} = r \left[\left(\sin\theta\cos\alpha + \frac{\sqrt{2}}{2} \cos\theta\sin\alpha \right)^{2} + \left( \frac{\sqrt{2}}{2}\cos\theta \cos\alpha - \sin\theta\sin\alpha \right)^{2} \right]^{\frac{1}{2}} \\ = r \left(\sin^2\theta + \frac{1}{2}\cos^2\theta\right)^{1/2} \end{align}

During the planning process, the user must manually select collimator position, size (or collimator diameter at the isocenter), and path density. In our facility, we have path densities of 3, 4, 5, 6, 8, 10, and 17. What the user selects, when a certain path density is chosen, is the number of arcs of constant oblique angle, but those oblique angles vary depending on the isocenter position.

Solid angle covered by the source

**Figure 7:**
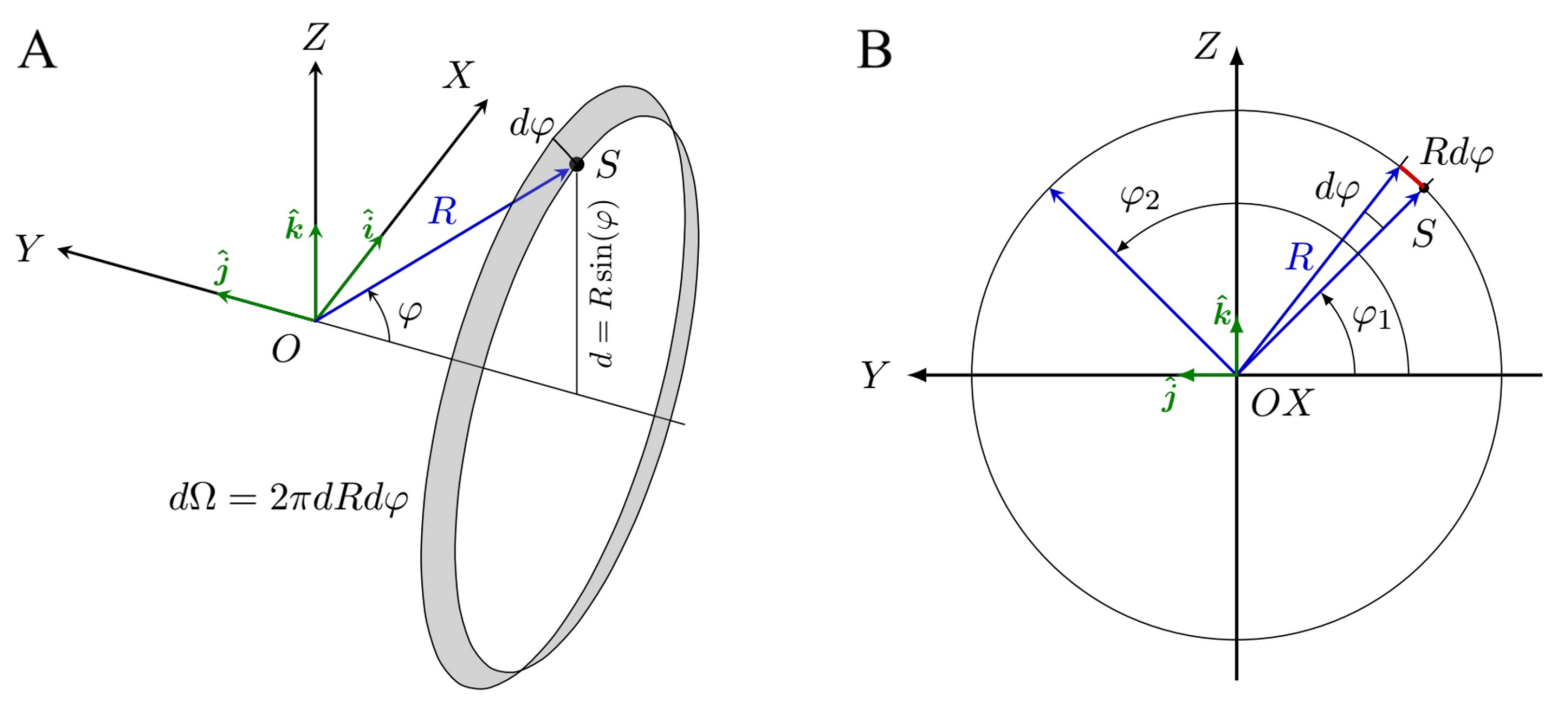
Custom spherical coordinate system used to calculate the solid angle covered by the source (A) Spherical coordinate system, orthogonal axis system, and differential element of area used for the calculation of the theoretical geometric efficiency of ZAP-X and Gamma Knife. (B) φ angle extension from φ1 to φ2

The solid angle covered by the source can be easily calculated using a custom spherical coordinate system (Figure 7). The point $O$​​​​​​​​​​​​​​ is the origin of coordinates, placed at the isocenter. The $OX$​​​​​​​​​​​​​​ axis is the right-to-left direction; the $OY$​​​​​​​​​​​​​​ axis, the foot-to-head direction; and the $OZ$​​​​​​​​​​​​​​ axis, the back-to-front direction (it is the same coordinate system as the one described in section "General geometric description of the gyroscopic system"). The $\varphi$​​​​​​​​​​​​​​ angle is defined as the angle between the $XY$​​​​​​​​​​​​​​ plane and the vector $R$​​​​​​​​​​​​​​ within the $YZ$​​​​​​​​​​​​​​ plane, being $R$​​​​​​​​​​​​​​ the vector connecting the isocenter, $O$​​​​​​​​​​​​​​; and $S$​​​​​​​​​​​​​​, the radiation source. A differential element of area covered by the source, $d\Omega$​​​​​​​​​​​​​​, can be expressed as

\begin{align} d\Omega = 2 \pi d \ R d\varphi \end{align}

and therefore, the total solid angle covered by the source will be given by

\begin{align} \Omega = \int_{\varphi_1}^{\varphi_2} d\Omega \end{align}

ZAP-X

In the case of ZAP-X, the angle $\varphi$​​​​​​​ will be between

\begin{align} \varphi_1 = \pi/4 \leqslant \varphi \leqslant \varphi_2 = 3\pi/4 \end{align}

Substituting $d = R\sin\theta$​​​​​​​ and integrating, we can calculate the theoretical solid angle covered by the source, $\Omega$​​​​​​​, as

\begin{align} \Omega = \int_{\varphi_1}^{\varphi_2} d\Omega = \int_{\pi/4}^{3\pi/4} 2 \pi R^2 \ \sin\varphi \ d\varphi = 2 \pi R^2 [-\cos\varphi]_{\pi/4}^{3\pi/4} = 2 \pi R^2 \ \sqrt{2} \end{align}

Therefore, the maximum theoretical geometric efficiency that we can achieve with the ZAP-X, $\eta_t$​​​​​​​, can be calculated as

\begin{align} \eta_t = \frac{\Omega}{4\pi R^2} =\frac{2 \sqrt{2} \ \pi R^2}{4\pi R^2} = \frac{\sqrt{2}}{2} = 0.71 \end{align}

In other words, it is possible to cover 71% of the surface around the patient’s head.

Gamma Knife Perfexion

In the case of Gamma Knife Perfexion, the geometric efficiency can be calculated in the same way, but considering that the $\varphi$​​​​​​​ angle is between

\begin{align} \varphi_1 \sim 47.5 ^{\circ} \leqslant \varphi \leqslant \varphi_2 \sim 87.0 ^{\circ} \end{align}

These values can be determined from a cutaway of the beam channels in the Gamma Knife Perfexion [7]. It must be taken into account that the Perfexion’s collimator body is conical, not hemispheric. Therefore, each row of sources has a different source to isocenter distance, so the $\varphi_1$​​​​​​​​​​​​​​ and $\varphi_2$​​​​​​​​​​​​​angles can be slightly affected by this distance. The theoretical solid angle covered by the source and the maximum theoretical geometric efficiency will be given, respectively, by

\begin{align} \Omega = \int_{\varphi_1}^{\varphi_2} d\Omega = 2 \pi R^2 [-\cos\varphi]_{\varphi_1}^{\varphi_2} = 2 \pi R^2 \ 0.62 \end{align}

\begin{align} \eta_t = \frac{\Omega}{4\pi R^2} =\frac{1.2465 \ \pi R^2}{4\pi R^2} = 0.31 \end{align}

Materials and methods

The ZAP-X is a dedicated, self-contained, and self-shielded GRS with a 3 MV LINAC mounted within a combination of yoked gimbals, allowing $2\sqrt{2}\pi$​​​​​​​ steradian solid angle beam coverage. Although irradiation is possible from any angle in the TDS with an accuracy of 0.1°, in the TPS, the nodes are distributed equally spaced over the circumferences every 6°, giving rise to 60 different positions in the axial gantry (from 0 to 354 in 6° steps) and 31 in the oblique (from 0 to 180 in 6° steps), resulting in a total of 1860 available nodes (60 x 31 = 1860). This set of nodes has been defined by Zap. Therefore, beams can be delivered from 1860 defined directions (Figure 6), but a given treatment will only use beams from a small subset of these directions.

During the planning process, the TPS does not allow users to select the nodes they wish to use (select axial and oblique gantry positions). Direct planning is not possible. It also does not allow users to visualize planned node positions until a treatment or fraction has been delivered.

The TDS allows the operator to collect data from all delivered treatments using an easy-to-use treatment reporting tool accessible from the TDS. All data collected corresponds to a sample of more than 200 patients treated at our facility over a two-year period, from October 2022 to April 2025, for various conditions: trigeminal neuralgias, obsessive-compulsive disorders, arteriovenous malformations, cavernomas, meningiomas, glioblastomas, schwannomas, multiple metastasis, etc., and with treatment volumes of up to 60 cubic centimeters. All treatment plans were designed and optimized with the ZAP-X TPS in its DP1010 version and previous versions.

## Discussion

**Figure 8:**
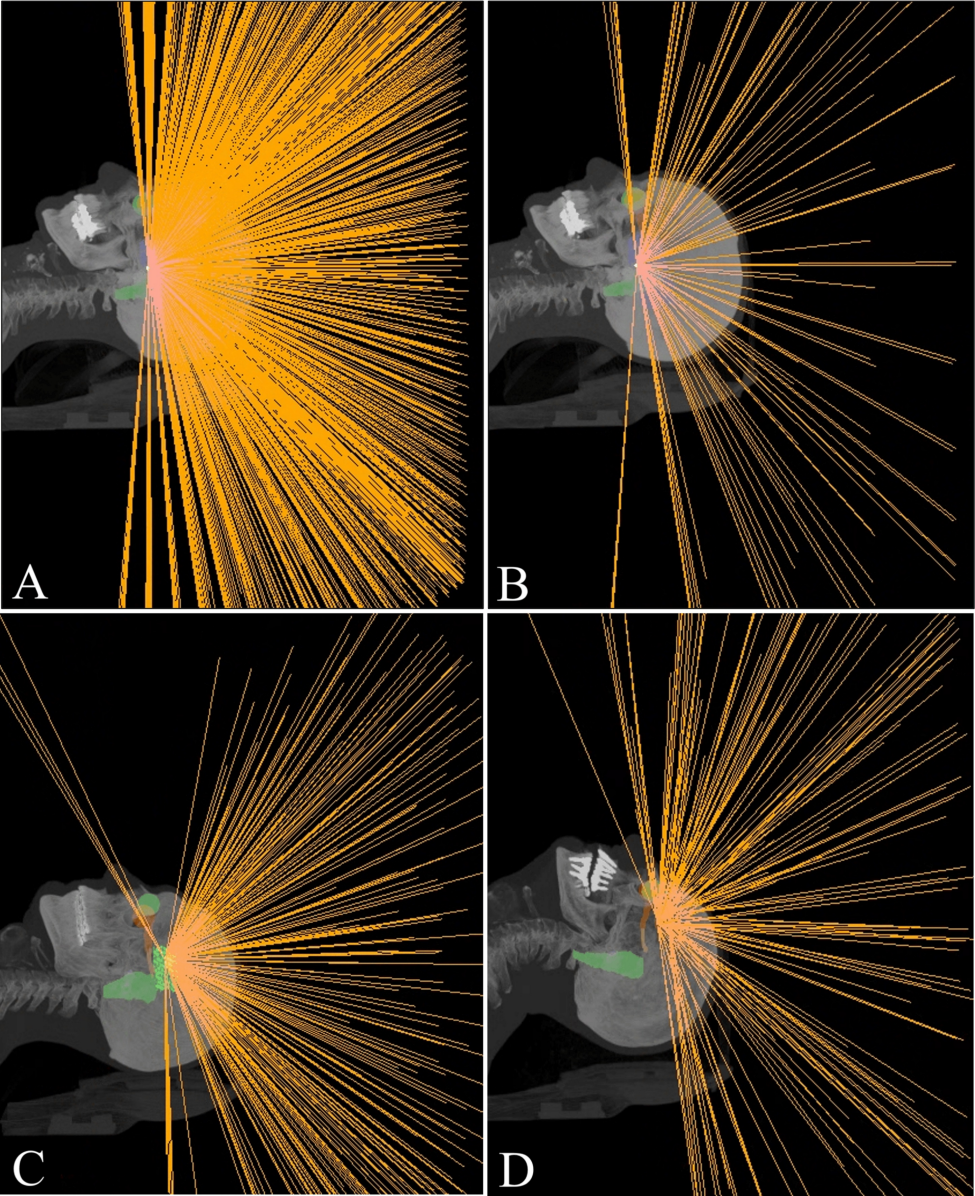
3D view from the ZAP-X Treatment Planning System (TPS) (A) The user can visualize all available nodes for a path density of 17, the maximum possible. Each orange line corresponds to a node and shows the path of the radiation beam to the isocenter. It can be clearly seen that oblique angles less than 84° are not allowed in any case (A and B images). (B) All available nodes for a path density of 5. (C) Clinical case in which nodes with a gantry angle less than 84º were used: an obsessive-compulsive disorder. (D) Clinical case in which nodes with a gantry angle less than 84º were used: a meningioma

The geometric performance that the ZAP-X can achieve in clinical practice is different from and smaller than the geometric efficiency of the system. In all treatments, our system has used 1026 different nodes, 55.1% of all available (Figure 6), resulting in a geometric performance of $0.551\cdot 2\sqrt{2} \pi R^2 = 1.558 \pi R^2$. It can be seen that oblique angles less than 84° are rarely used and correspond only to two treatments delivered after the last update (Figure 8).

The comparison between the clinical geometric performance and the theoretical geometric efficiency of ZAP-X and Gamma Knife is shown in Table 2. While ZAP-X still does not get the most out of its technical characteristics, Gamma Knife obtains full performance. However, geometric efficiency and clinical performance are higher in ZAP-X.

**Table 2:** Comparison between the clinical geometric performance and the theoretical geometric efficiency of ZAP-X and Gamma Knife

Description	ZAP-X	Gamma Knife
Maximum solid angle covered by the source, Ω	2√2πR^2 ^= 2.83 πR^2^	1.25 πR^2^
Theoretical geometric efficiency, η_t_	71%	31%
Clinical solid angle covered by the source, Ω_clin_	1.56 πR^2^	1.25 πR^2^
Clinical geometric performance, η_clin_	39%	31%

## Conclusions

The ZAP-X system, due to its design based on a combination of yoked gimbals, has a greater geometric efficiency and clinical geometric performance than the Gamma Knife system. Despite the fact that ZAP-X limits the minimum oblique angle to $84^{\circ}$ in most cases, what can be understood as a conservative choice due to the reduced amount of available workspace at the lower oblique angles (the patient’s shoulders and the table limit the collision-free space, so they have specifically limited where they allow beams), there is still a lot of room for improvement that can lead to this new gyroscopic radiosurgery system beyond its current limits.
